# Neuromuscular Electrical Stimulation Does Not Influence Spinal Excitability in Multiple Sclerosis Patients

**DOI:** 10.3390/jcm13030704

**Published:** 2024-01-25

**Authors:** Martina Scalia, Riccardo Borzuola, Martina Parrella, Giovanna Borriello, Francesco Sica, Fabrizia Monteleone, Elisabetta Maida, Andrea Macaluso

**Affiliations:** 1 Department of Movement, Human and Health Sciences, University of Rome “Foro Italico”, 00135 Rome, Italy; m.scalia@studenti.uniroma4.it (M.S.); r.borzuola@studenti.uniroma4.it (R.B.); m.parrella@studenti.uniroma4.it (M.P.); andrea.macaluso@uniroma4.it (A.M.); 2 Neurology Unit, San Pietro Fatebenefratelli Hospital, MS Centre, 00189 Rome, Italy; 3 Santa Maria Goretti Hospital, 04100 Latina, Italy; f.sica@ausl.latina.it (F.S.); f.monteleone@ausl.latina.it (F.M.); 4 Department of Advanced Medical and Surgical Sciences, University of Campania “Luigi Vanvitelli”, 80138 Naples, Italy; maidaelisabetta92@gmail.com

**Keywords:** electric stimulation, H-reflex, spinal excitability, multiple sclerosis, soleus, GABA, presynaptic inhibition, rehabilitation

## Abstract

(1) **Background**: Neuromuscular electrical stimulation (NMES) has beneficial effects on physical functions in Multiple sclerosis (MS) patients. However, the neurophysiological mechanisms underlying these functional improvements are still unclear. This study aims at comparing acute responses in spinal excitability, as measured by soleus Hoffmann reflex (H-reflex), between MS patients and healthy individuals, under three experimental conditions involving the ankle planta flexor muscles: (1) passive NMES (pNMES); (2) NMES superimposed onto isometric voluntary contraction (NMES+); and (3) isometric voluntary contraction (ISO). (2) **Methods**: In total, 20 MS patients (MS) and 20 healthy individuals as the control group (CG) took part in a single experimental session. Under each condition, participants performed 15 repetitions of 6 s at 20% of maximal voluntary isometric contraction, with 6 s of recovery between repetitions. Before and after each condition, H-reflex amplitudes were recorded. (3) **Results**: In MS, H-reflex amplitude did not change under any experimental condition (ISO: *p* = 0.506; pNMES: *p* = 0.068; NMES+: *p* = 0.126). In CG, H-reflex amplitude significantly increased under NMES+ (*p* = 0.01), decreased under pNMES (*p* < 0.000) and was unaltered under ISO (*p* = 0.829). (4) **Conclusions**: The different H-reflex responses between MS and CG might reflect a reduced ability of MS patients in modulating spinal excitability.

## 1. Introduction

Multiple sclerosis (MS) is the most common immune-mediated disorder that affects the central nervous system (CNS) [1], involving approximately 2.8 million people worldwide [2]. MS is characterized by axonal damages in the brain and the spinal cord, which lead to conduction block or a delay of electrical potentials along neuronal pathways throughout the CNS [3,4]. Consequently, people with MS (pwMS) experience a wide range of disabilities [5,6], which lead to a progressive limitation of functioning in daily activities, thus reducing the health-related quality of life (HRQOL) [7,8].

In MS rehabilitation, physical activity is an important non-pharmacological tool for countering the multifaceted symptoms of the disease [9,10,11]. The benefits of exercise for the MS population include improving or maintaining walking ability and balance, cardiovascular and neuromuscular fitness, physical and psychological fatigue, HRQOL, depression, and chronic disease risk profiles [12,13,14,15,16,17,18,19,20]. Unfortunately, despite the benefits of physical activity, 78% of pwMS are physically inactive [11,21,22]. Particularly, patients with advanced MS, or wheelchair users, may find exercise very difficult due to significant fatigue, leg muscle paresis, and poor flexibility [23]. For these reasons, several rehabilitative techniques have been developed in recent years to improve motor function using technological devices, such as neuromuscular electrical stimulation (NMES). NMES is a Food and Drug Administration-approved treatment for reducing muscle pain and spasm, as well as disuse-associated muscle atrophy [24]. NMES induces visible muscle contractions by depolarizing local motor nerves via intermittent electrical stimuli that are transcutaneously applied to superficial skeletal muscles [25,26]. This training technique has been widely employed in rehabilitation clinics to treat several pathological conditions of the neuromuscular system [27,28,29] and other neurological diseases [30,31]. Furthermore, many researchers have reported a broad range of positive effects of NMES on motor and brain functions [29,31,32,33]. Specifically, in relation to MS, some evidence suggests that individuals with greater levels of disability can experience gains in physical function and perceived physical health when exercise training is combined with NMES [34,35]. Some authors found that supplementing a traditional training program with NMES improves muscle strength, force steadiness, gait speed, walking endurance, self-reported levels of walking disability, and balance [36,37,38], as well as reducing fatigue levels in pwMS, even in those who use walking aids [38]. Furthermore, other studies have reported that NMES applied during cycling enhances muscle strength and cardiorespiratory metabolism, improves walking, and increases the ability to transfer independently for individuals who rely on manual wheelchairs for daily mobility [34,35]. Altogether, these findings suggest that NMES could be considered a promising treatment option for persons with primary or advanced MS, who often have a very limited capacity for strength and aerobic exercise. However, although these clinical results have been recognized by researchers and clinicians, the neurophysiological mechanisms behind NMES’s training benefits are still unclear.

The Hoffmann reflex (H-reflex), which is a spinal reflex elicited by electrically stimulating a peripheral mixed nerve, has been extensively used to investigate some of the neurophysiological mechanisms underlying NMES intervention in both healthy young and elderly individuals [39,40,41,42,43]. As a monosynaptic spinal reflex, the H-reflex could offer an effective tool for studying the modulation of spinal excitability under certain conditions [44]. Several research works have revealed that NMES stimulation modulates H-reflex responses in healthy individuals [39,41,42,43,45,46]. Some authors have demonstrated that a single bout of NMES superimposed onto voluntary isometric contractions of the ankle plantar flexor muscles increased soleus H-reflex amplitude [39,40,41]. On the contrary, recent evidence has indicated a sudden reduction in H-reflex amplitude following passive NMES [39,41,43,47,48,49]. Based on these results, several authors have stated that NMES modulates spinal excitability in healthy individuals by acting on some presynaptic mechanisms, which are mainly involved in the facilitation/inhibition of the H-reflex [39,41,42,43,47,50]. However, to the best of our knowledge, no studies have investigated these mechanisms after NMES intervention in neurological populations, particularly in pwMS.

Therefore, the aim of this study was to compare spinal reflex responses, as measured by the H-reflex of the Soleus (SOL) muscle, between MS patients and healthy individuals, following a single intervention consisting of three experimental conditions: (1) NMES superimposed onto voluntary contraction (NMES+) of the plantar flexor muscles of the ankle; (2) passive NMES (pNMES) applied to the plantar flexor muscles of the ankle; (3) voluntary isometric contractions (ISO) of the plantar flexor muscles of the ankle. Based on the results reported in previous studies that have applied this protocol to healthy individuals [39,41], the first hypothesis is that the H-reflex would increase after NMES+ in MS patients and healthy individuals; the second hypothesis is that the H-reflex would decrease after pNMES in MS patients and healthy individuals; the third hypothesis is that the H-reflex would remain unchanged after the ISO condition in MS patients and healthy individuals.

## 2. Materials and Methods

### 2.1. Participants

In total, 40 volunteers participated in the study and were divided into 2 groups: “MS”, which involved 20 patients with MS (mean ± SD, age: 38.8 ± 10.9 years, mass: 68.8 ± 13.1 kg, height: 1.72 ± 0.8 m; EDSS < 5); “CG”, which involved 20 healthy individuals as the control group (mean ± SD, age: 39.2 ± 11.9 years, mass: 67.8 ± 12.26 kg, height: 1.71 ± 0.7 m). Statistical power analyses (G*Power software v.3.1.9.4) for a mixed-model ANOVA (within–between factors) were conducted a priori to determine the sample size (α = 0.05, effect size = 0.26, statistical power = 0.95) [51]. The sample size estimation is based on evidence from previous neurophysiological studies exploring the immediate effect of NMES on H-reflex amplitude in healthy young and older individuals [39,41,43], as well as previous studies investigating the H-reflex in pwMS [52,53].

MS participants were recruited according to the following inclusion criteria [36,53]: relapsing–remitting MS, EDSS score ≤ 5, aged between 20 and 60 years, able to walk independently at household distances, never had experience with NMES before. On the other hand, they were excluded from the study according to the following exclusion criteria [36,53]: MS relapse in the past three months; comorbidity with other disorders, such as cardiovascular disease, orthopedic conditions involving bone fractures in the lower limbs, history of seizures or/and epilepsy, neural lesions at or below the lumbar enlargement, and lung disorders; contraindications to electrical stimulation, such as implanted pacemaker or other biomedical devices or metal, allergies to surface electrode gel, and ongoing pregnancy. CG participants were recruited by matching them with the MS patients for age, gender, height, and body mass. They were excluded from the study if they presented any neurological or orthopedic disorders. In addition, both MS and CG volunteers were recruited if they were physically inactive [54,55], as physical activity levels, or specific sport practice, could influence spinal excitability [56].

MS participants were recruited at the Multiple Sclerosis Centers of “Santa Maria Goretti” Latina hospital and “San Pietro, Fatebenefratelli” Rome hospital, and were identified using an initial screening questionnaire that was administered by the clinicians. The entire sample of recruited pwMS had an EDSS score ranging between 1 and 3, which corresponds to mild disability with no or minimal impairment of ambulation [57]. However, eleven of the recruited patients had spinal cord lesions due to MS; therefore, the level of pain in all patients was clinically tested with the Visual Analog Scale (VAS) as well as the presence or absence of spasticity with the Modified Ashworth Scale (MAS). As for the VAS, only 5 out of 20 patients reported pain (score 1) due to paresthesia and low back pain; the other 15 patients did not report pain (score 0). As for the MAS, the scores also showed no spasticity and no limitation of the range of motion. In the light of these data, it can be concluded that the whole sample did not present detectable pyramidal signs, disabilities or symptoms related to spasticity. Patients’ data are illustrated in Table 1.

CG participants were recruited at the University of Rome—Foro Italico and were identified by the research assistant using an initial screening questionnaire. During a first appointment, participants of both groups received the participant information sheet and were verbally informed of the study procedures. They were shown the equipment and were given 15 min to familiarize themselves with NMES. During a second appointment (2–4 days later) they were asked to sign an informed consent that was approved by the institutional ethics review board of the University of Rome—“Foro Italico” (CAR 120/2022). Participants who met the inclusion/exclusion criteria and gave informed consent were assigned to one of the two groups (MS versus CG), according to the presence of the disease.

### 2.2. Instrumentation

#### 2.2.1. Maximum Voluntary Isometric Contraction (MVIC)

**Figure 1:**
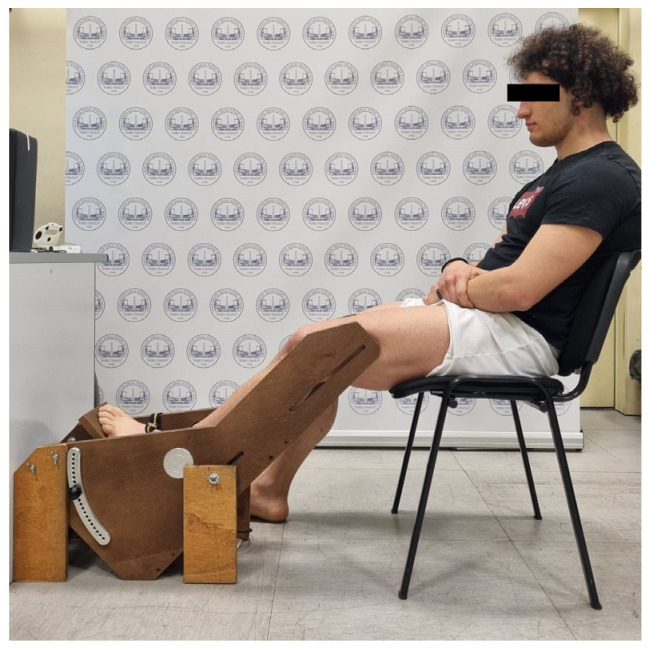
Participant’s position. Participant’s position on the dynamometer chair during the MVIC, the H-reflex recruitment curve (RC) and the three experimental conditions (pNMES, NMES+, ISO).

An ankle dynamometer v1.0 (OT Bioelettronica, Turin, Italy) was used to measure maximal voluntary isometric contractions (MVIC) of the ankle plantar flexor muscles. Participants were asked to sit in a standard position [39,41], with hips at 90° (0° = neutral hip position), knees at 60° (0° = full knee extension) and ankles at 0° of ankle plantar–dorsiflexion (0° = foot orthogonal to the shank axis) [39,41] as described in Figure 1. In this position, the foot was firmly secured with two bands to the footplate of the dynamometer, with the lateral malleolus aligned with the dynamometer’s axis. Warm-up and familiarization exercises consisted of 15–20 submaximal isometric contractions. MVIC testing involved a rapid increase in ankle plantar flexor force to a maximum. A computer screen provided visual feedback to participants during the test. During the MVIC assessment, participants received verbal encouragement and were asked to maintain their maximal isometric contraction for at least three seconds before relaxing. The best result was selected after a minimum of three attempts, separated by 3 min rest intervals to minimize muscle fatigue. During each experimental condition, a target force of 20% of MVIC was set based on MVIC measurements. Previous studies involving the application of NMES demonstrated that this force level could affect spinal excitability without inducing muscle fatigue [39,40,41,42,48,58]. In this regard, MVIC was evaluated again at the end of the whole experimental session to determine if muscle fatigue had arisen.

#### 2.2.2. Surface Electromyography (sEMG)

**Figure 2:**
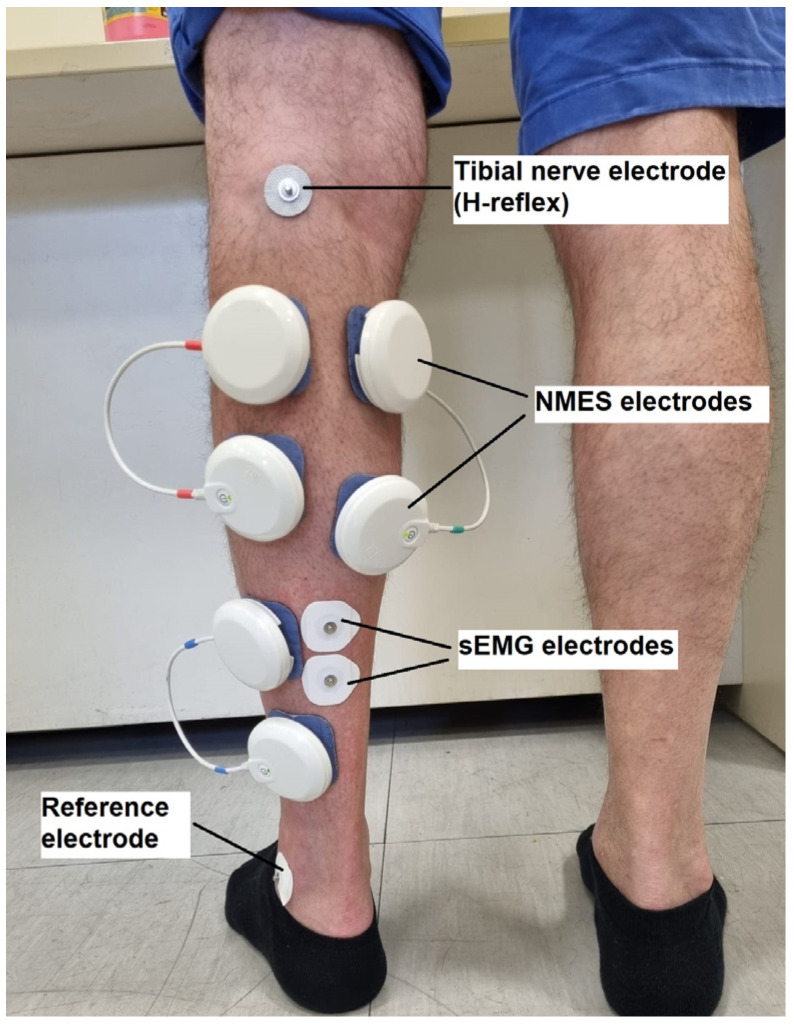
Positioning of electrodes. Wireless neuromuscular stimulator electrodes (white pods) are placed bipolarly on the gastrocnemius medialis, gastrocnemius lateralis, and soleus muscles. Two sEMG electrodes are placed in a bipolar configuration on the soleus muscle and one reference electrode is positioned on the lateral malleolus. The electrode for the electrical stimulation of the H-reflex electrode is placed on the posterior tibial nerve.

Muscle activation was measured via surface electromyography (sEMG) using a 64-channel EMG device with Wi-Fi communication (OT Bioelettronica, Turin, Italy; sampling frequency 2000 Hz). Two electrodes (36 × 40 mm, FIAB, Florence, Italy; inter electrode distance: 20 mm) were placed in a bipolar configuration on the SOL muscle, about 2 cm below the gastrocnemius myotendinous junction [56]. An additional reference electrode (36 mm × 40 mm, FIAB, Florence, Italy) was placed on the lateral malleolus (Figure 2). To attach the electrodes properly and ensure an impedance below 5 kΩ, the skin of the participants was first softly rubbed and cleaned.

#### 2.2.3. Soleus H-Reflex

**Figure 3:**
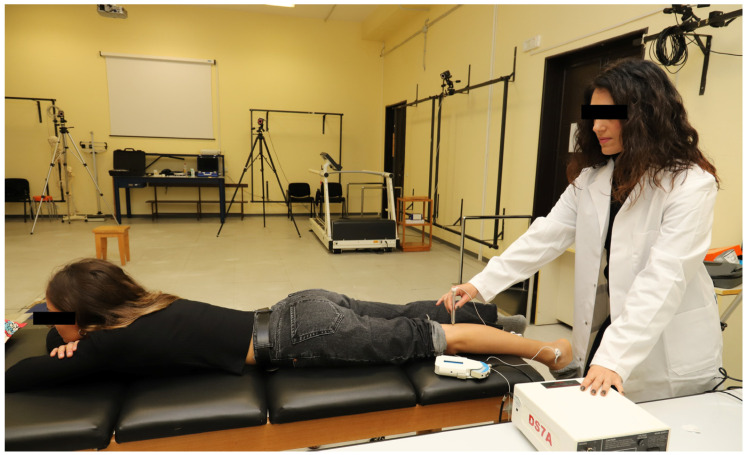
Participant’s position during the H-reflex assessment. Participant’s prone position on the physiotherapy bed while the investigator located the optimal stimulation site in the popliteal fossa using a cathode ball electrode to evoke the H-reflex of the SOL muscle.

**Figure 4:**
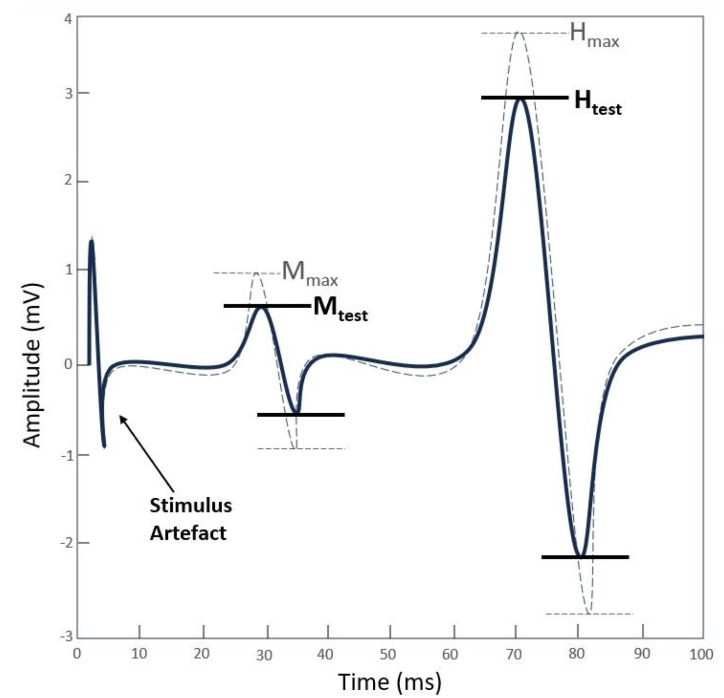
Procedure for selection of H−reflex and M−wave. The grey dashed line represents the maximum H−reflex (H_max_) and the corresponding M−wave (M_max_). The black solid line represents the H−reflex test (H_test_) (80−85% of H_max_) and the corresponding M−wave test (M_test_) (5% of M_max_).

Single rectangular biphasic pulses of 1 ms duration were delivered to the posterior tibial nerve using a constant-voltage electrical stimulator (Digitimer Ds7a, Digitimer Ltd., Hertfordshire, UK). As a first step, participants were asked to lie down on a physiotherapy bed in a prone position to find the optimal stimulation site in the popliteal fossa, which was identified using a cathode ball electrode, as illustrated in Figure 3. Therefore, to elicit the H-reflex, one cathode electrode (diameter 24 mm, Spes Medica, Genova, Italy) was placed over the posterior tibial nerve, and one anode electrode (diameter 50 × 50 mm, Compex Dura-Stick^®^ Plus a Snap, DJO Global, Vista, CA, USA) was placed above the patella [39,41,56]. Afterwards, participants were asked to sit in the dynamometer chair, as described in the MVIC section (Figure 1). In this position, the H-reflex recruitment curve (RC) was assessed using sEMG to record the involuntary muscle activity of the triceps surae muscle in response to the stimulation. The RC was obtained by plotting the amplitude of the recorded H-reflexes over the amplitude of the corresponding motor waves (M-waves). Based on previously established procedures [39,41,56], the tibial nerve was stimulated with a series of single electrical stimulations, gradually increasing the current intensity. A stimulation interval of 4–10 s was chosen to prevent fatigue [59], with stimulus intervals unevenly spaced to prevent anticipation and reduce post-activation depression [59]. The stimulus intensity was gradually increased with steps of 1 mA until the maximum H-reflex (H_max_) and motor wave (M_max_) amplitudes were reached; to confirm no further increase in M_max_ amplitude, stimulus intensity was increased slightly beyond this point. A peak-to-peak analysis of the sEMG recordings was used to measure the amplitude of H-reflexes and M-waves. According to previous studies [39,41,56], the stimulus intensity was selected to evoke an H-reflex (H_test_) on the ascending limb of the RC with an amplitude corresponding to 80–85% of the Hmax, as illustrated in Figure 4. To ensure stimulus consistency and repeatability, a small M-wave (M_test_) corresponding to the H_test_ was selected and monitored throughout the entire experiment [39,40]. If the M-wave amplitude lay within 5% of the selected M_test_, the corresponding H-reflex measure was accepted. Each experimental condition was preceded and followed by the assessment of 20 H-reflexes. Additionally, the amplitudes of all H-reflexes and M-waves were normalized to M_max_ and averaged within each trial. Furthermore, the H_max_/M_max_ ratio of both MS and CG was calculated, using the amplitudes measured during the RC, to determine the overall level of reflex excitability of the motor pool at rest.

For this study, the H-reflex of the SOL muscle was chosen given the accessibility of the posterior tibial nerve, which could be easily identified and stimulated to evoke the reflex responses. Moreover, as the SOL muscle has stronger spinal connections compared to other limb muscles [42,60], the SOL H-reflex has been one of the most widely studied reflexes in assessing spinal excitability [39,40,41,56].

#### 2.2.4. Neuromuscular Electrical Stimulation (NMES)

A muscle stimulator (Chattanooga Wireless Professional, DJO Global, Vista, CA, USA), which produces rectangular, balanced biphasic pulses, was used to electrically stimulate the ankle plantar flexor muscles. The NMES stimulator was always accurately and safely managed by the investigator. At the beginning of the experimental session, the motor points of the gastrocnemius lateralis, gastrocnemius medialis, and soleus muscles were determined using a hand-held cathode ball electrode, as described in the electrical stimulator user’s guide. Here, 3 self-adhesive electrodes (diameter 50 × 50 mm, Compex Dura-Stick^®^ Plus a Snap, DJO Global, Vista, CA, USA) with positive polarity were applied to the motor points of the three muscles. Hence, an electrode with negative polarity was placed about 3 cm above each positive electrode (Figure 2). A pulse frequency of 20 to 50 Hz and a pulse duration of 400 μs were selected to administer NMES. These parameters were chosen to reduce discomfort during NMES [25]. The operator gradually increased the stimulation intensity until the muscle contraction intensity reached 20% of MVIC, either with passive or superimposed NMES. According to Wiest et al. (2017) [61], an NMES intensity that generates 20% of MVIC force does not cause pain or discomfort.

### 2.3. Experimental Procedure

All measurements were conducted in the “Laboratory of Bioengineering and Neuromechanics of Movement” at the University of Rome “Foro Italico” between November 2022 and May 2023. Each participant took part in a single experimental session lasting around 150 min. First, participants lay prone on a physiotherapy bed while the investigator located the correct point for electrical nerve stimulation in the popliteal fossa (Figure 3) as well as the motor points on the triceps surae for NMES. Then, the participants were familiarized with the NMES for 10 min. After that, they were seated on the dynamometric chair, as previously described (Figure 1), and maintained this position for the entire experimental session. Visual feedback was provided by a computer screen. In this position, the MVIC of the triceps surae muscles as well as the H-reflex RC were assessed [39,41]. Following this, participants were exposed to three different experimental conditions that involved ankle plantar flexor muscles: (1) passive NMES (pNMES); (2) NMES superimposed onto voluntary isometric contraction (NMES+); (3) voluntary isometric contraction only (ISO).

**Figure 5:**
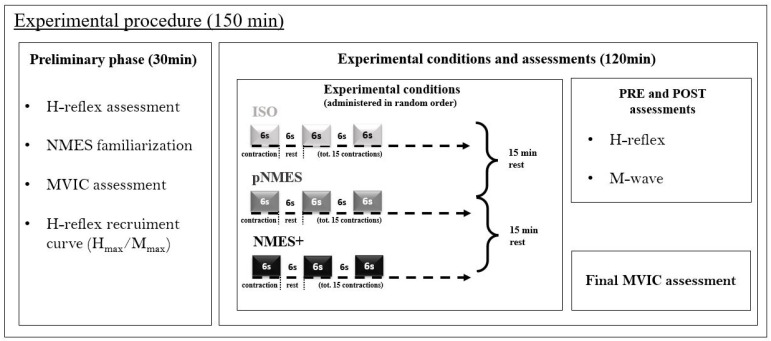
Experimental protocol diagram. This is an overview of the measurements performed during the preliminary phase, as well as the description of the three experimental conditions.

The order of conditions was randomly administered to each participant. During each condition, which lasted around 3 min, participants were asked to perform 15 intermittent contractions (6 s contraction/6 s rest). To prevent long-lasting effects caused by the previous condition, recovery periods of 15 min were enforced between the conditions [39,40,62]. The entire experimental protocol, illustrated in Figure 5, was designed to modulate spinal excitability, and to prevent muscle fatigue, as reported in previous investigations [39,41,49]. In the ISO condition, participants were instructed to contract their plantar flexor muscles voluntarily to achieve 20% of MVIC. In the pNMES condition, passive stimulation was applied to the plantar flexor muscles to reach 20% of MVIC. Participants were instructed not to voluntarily contract their ankle plantar flexor muscles during the passive NMES to isolate the effects of NMES intervention. To the best of our knowledge, none of the participants voluntarily contracted their muscles during pNMES. In the NMES+ condition, current pulse intensity was set to produce half of the target force (10% of MVIC), while participants voluntarily contracted their plantar flexor muscles at 10% of MVIC to reach the full target force (20% of MVIC). The investigator adjusted the intensity of the simulation by asking participants to relax their calf muscles before and after the first and the tenth contractions. Whenever participants reported pain or discomfort, NMES conditions were immediately stopped. The H-reflex was measured at baseline (PRE) and at the end of each experimental condition (POST). To exclude the contribution of fatigue, the MVIC was repeated at the end of the entire experiment. For MS, all procedures were performed on the participant’s weaker or more affected leg (based on self-report) [36]. For CG, all procedures were performed on the participant’s dominant leg [26], which was determined as the preferred limb for hopping or kicking a ball [63].

### 2.4. Data Analysis

All data were analyzed using a custom Matlab code (Matlab 2018b, Mathworks Inc., Natick, MA, USA). The sEMG recordings were checked for possible pre-activation of the SOL muscle before reflex assessment. If pre-activation occurred, the sEMG trace and the associated H-reflex measure were removed from the analysis.

### 2.5. Statistical Analysis

Statistical analysis was performed using IBM SPSS 24.0 (IBM Corp., Armonk, NY, USA). A two-way mixed ANOVA was used to investigate statistical differences in H-reflex and M-wave measures between the two groups, the three experimental conditions, and over time. “Condition” and “Time” represented the two within-subjects factors, with “Condition” having three levels (ISO, pNMES and NMES+) and “Time” having two levels (PRE and POST). “Group” represented the between-subjects factor, with “MS” referring to MS patients, and “CG” to healthy individuals of the control group. When a significant main effect or interaction was found, paired *t* tests were used for post hoc analyses. In addition, a *t* test was performed to compare MVIC values at the beginning (Pre-test) and at the end (Post-test) of the entire experimental protocol in both MS and CG, as well as to compare the baseline level of the H_max_/M_max_ ratio between the two groups. The alpha level for statistical significance was set to *p* < 0.05, with a Bonferroni correction for multiple post hoc comparisons. The normality and sphericity of the data were checked using the Shapiro–Wilk Test and the Mauchly Test, respectively. Data are reported as group mean ± standard deviation (SD).

## 3. Results

All the recorded data showed a normal distribution, and the Mauchly test confirmed that the assumption of sphericity was not violated for any of the variables analyzed.

The mixed ANOVA on the normalized H-reflex amplitude showed a main effect of Condition (F = 3.292, ηp2 = 0.017, *p* = 0.043) and a Condition*Group interaction (F = 4.909, ηp2 = 0.025, *p* = 0.010), a Condition*Time interaction (F = 16.137, ηp2 = 0.036, *p* < 0.000), and a Condition*Time*Group interaction (F = 9.569, ηp2 = 0.022, *p* < 0.000). Post hoc analysis showed no significant differences in H-reflex amplitude between PRE and POST measures for all experimental conditions (ISO: *p* = 0.506; pNMES: *p* = 0.068; NMES+: *p* = 0.126) in MS. Conversely, H-reflex amplitude significantly increased following NMES+ (+12.6%; *p* = 0.010), decreased after pNMES (−17.8%; *p* < 0.000), and was unaltered following ISO (*p* = 0.829) in CG. Moreover, post hoc analysis showed no significant differences in the amplitude of H-reflex measured before (PRE) all the three experimental conditions, in both MS (PRE ISO vs. POST ISO: *p* = 0.306; PRE pNMES vs. POST pNMES: *p* = 0.656; PRE NMES+ vs. POST NMES+; *p* = 0.339) and CG (PRE ISO vs. POST ISO: *p* = 0.342; PRE pNMES vs. POST pNMES: *p* = 0.076; PRE NMES+ vs. POST NMES+; *p* = 0.168). PRE and POST values of all conditions are reported in Table 2 as mean ± standard deviation (SD).

The mixed ANOVA on the normalized M-wave amplitude showed no effect of Time (F = 0.071, ηp2 = 0.002, *p* = 0.791) or Condition (F = 0.121, ηp2 = 0.003, *p* = 0.886) as well as no Condition*Group interaction (F = 0.119, ηp2 = 0.003, *p* = 0.888), no Time*Group interaction (F = 0.560, ηp2 = 0.015, *p* = 0.459), no Condition*Time interaction (F = 1.514, ηp2 = 0.038, *p* = 0.229) and no Condition*Time*Group interaction (F = 2.276, ηp2 = 0.057, *p* = 0.110).

**Figure 6:**
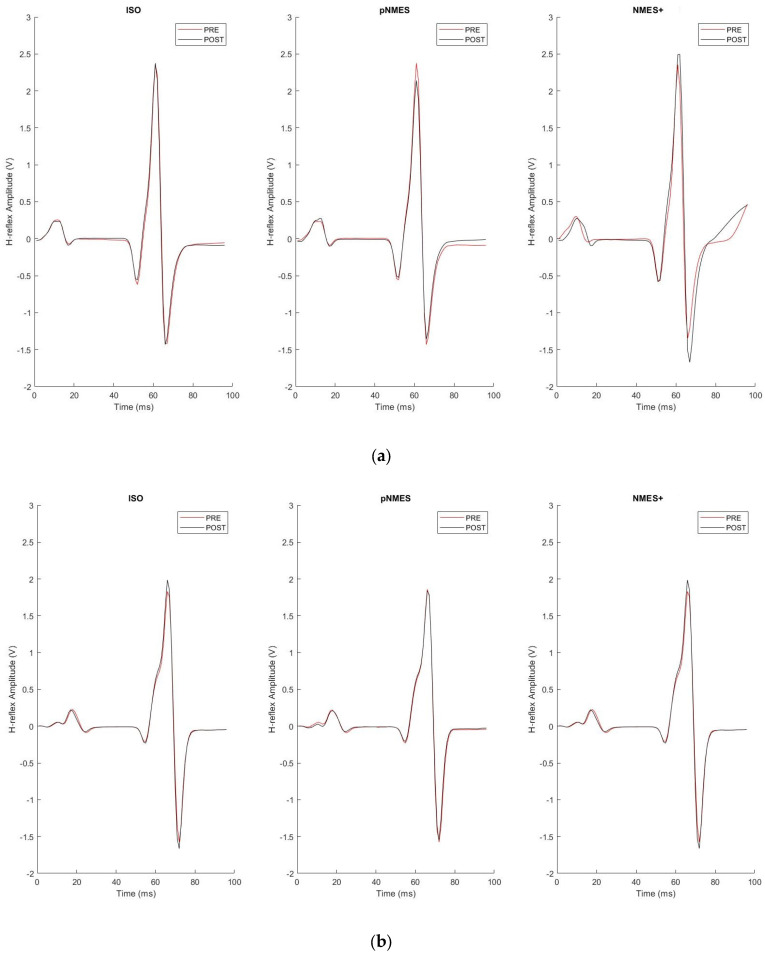
H−reflex and M−wave before and after the three experimental conditions. The figure shows an example of the difference in the mean of H−reflex responses following 20 electrical stimulations of the posterior tibial nerve, before (PRE) and after (POST) each experimental condition (ISO, pNMES, NMES+) in a patient of the MS group (**a**) and in a healthy participant of the GC (**b**). The red line represents the mean PRE; the black line represents the mean POST.

**Figure 7:**
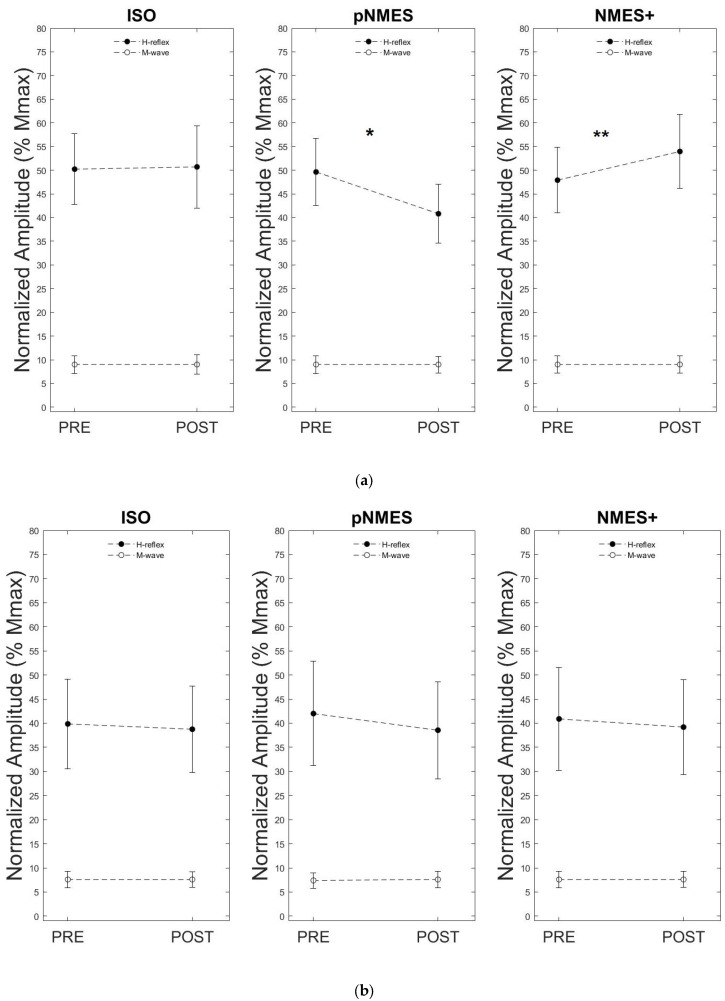
H-reflex and M-wave normalized by M_max_. (**a**) Amplitude of soleus H-reflexes and corresponding M-waves normalized to M_max_ before (PRE) and after (POST) the three experimental conditions (ISO, pNMES and NMES+), in CG. Data are reported as group means ± standard deviation (* *p* = 0.010; ** *p* < 0.000). (**b**) Amplitude of soleus H-reflexes and corresponding M-waves normalized to M_max_ before (PRE) and after (POST) the three experimental conditions (ISO, pNMES and NMES+) in MS. The H-reflex and M-wave amplitudes averages did not change in ISO, pNMES or NMES+. Data are reported as group means ± standard deviation.

Figure 6a,b describes a typical example of the SOL H-reflex and M-wave sEMG response to a series of 20 electrical stimuli that were averaged within the same trial before (PRE) and after (POST) each experimental condition (ISO, NMES, NMES+) in one healthy participant from the CG (Figure 6a) and in one MS participant from the MS (Figure 6b). Figure 7a,b reports the mean values of SOL H-reflex amplitude and associated M-waves that were both normalized to M_max_ before (PRE) and after (POST) the three experimental conditions (ISO, pNMES, NMES+) in CG (Figure 7a) and in MS (Figure 7b).

**Figure 8:**
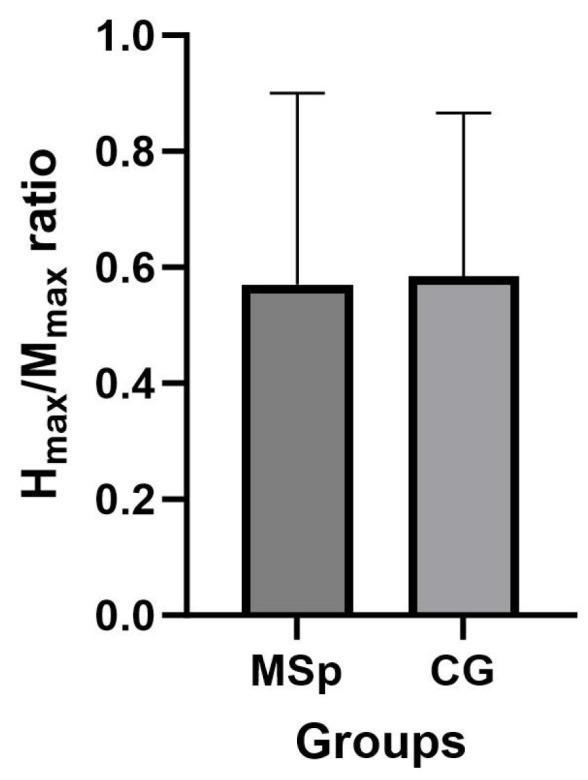
H_max_/M_max_ ratio. MS and CG showed no differences in the H_max_/M_max_ ratio values at rest.

The paired *t*-test analysis on the H_max_/M_max_ ratio showed no significant differences between MS and CG (*p* = 0.901), as illustrated in Figure 8.

The *t*-test analysis of the pre-test and post-test MVIC values showed no significant differences in both MS (*p* = 0.655) and CG (*p* = 0.267), as illustrated in Table 3.

## 4. Discussion

The main result of this study was the different H-reflex response between healthy individuals and MS patients following a single NMES session. According to our hypotheses, in healthy individuals the amplitude of H-reflex decreased after pNMES, increased after NMES+, and did not change after ISO, confirming that NMES modulates spinal excitability. However, in contrast with our hypotheses, in MS patients there were no significant differences in H-reflex amplitude following both pNMES and NMES+, thus suggesting an alteration in the control of some presynaptic mechanisms related to the modulation of spinal reflexes. Finally, as hypothesized, no significant differences were found in MS patients after ISO. Therefore, of clinical significance, it appears that in MS patients a single session of NMES, either passive or superimposed onto voluntary movement, has the same effect as voluntary isometric exercise and does not affect spinal excitability.

In our study, healthy individuals showed an acute attenuation of SOL H-reflex amplitude, which is consistent with the results of previous studies reporting that NMES inhibits spinal reflexes when it is passively applied [39,41,43,47,48,49]. These studies suggest that pNMES may induce specific neuroplasticity in the inhibitory pathway at the spinal cord level, leading to a decreased spinal excitability [43]. Indeed, several authors have stated that NMES directly acts on some presynaptic mechanisms that are primarily involved in the modulation of the H-reflex [47,56], such as presynaptic inhibition (PSI). PSI, which is one of the most important spinal regulatory networks [64], is mediated by the action of an inhibitory interneuron, which acts on the Ia-afferent terminals, leading to a reduction in the number of neurotransmitters released in the synapse between the Ia-afferent fiber and alpha-motoneuron. The decrease in neurotransmitter release results in a concomitant reduction in the depolarization of the alpha-motoneuron, which is induced by Ia-afferent activity. Therefore, it was proposed that increased PSI in Ia-afferent terminals could be considered as the main factor responsible for the substantial attenuation of soleus H-reflex amplitudes that are induced by passive NMES in healthy individuals [39,41,47,49,50]. A possible neurophysiological mechanism that may be associated with this phenomenon is primary afferent depolarization (PAD) [65], which induces a reduction in neurotransmitter release by previously depolarizing Ia-afferent terminals. Consequently, the neurotransmission between Ia-afferents and α-motoneurons is compromised. As suggested by Pierrot-Deseilligny and Mazevet (2000) [50], PAD interneurons might be more responsive to repetitive electrical stimulation due to their lower threshold, thus decreasing spinal excitability.

Regarding the effects of NMES+ on the healthy participants of our study, the increased SOL H-reflex amplitude was consistent with earlier studies in healthy subjects. Scalia et al. (2023) [41] and Borzuola et al. [39,40] reported an acute increase in H-reflex amplitude of between 5 and 20% after a single experimental session, during which participants were asked to voluntarily contract their ankle planta–flexor muscles while NMES was superimposed onto the same muscles. In addition, some authors observed similar responses in the H-reflex when electrical stimulation was applied to the tibial nerve during voluntary contractions of the ankle plantar flexion muscles [42]. Therefore, our results suggest that NMES+ may enhance spinal excitability, which might be associated with an increased force generation capacity in healthy individuals [42,56]. A reduced PSI in Ia-afferent terminals may be responsible for the significant increase in the H-reflex amplitude following NMES+ in healthy adults [39,41,47,56].

In contrast with our hypothesis, MS patients did not show any changes in the H-reflex responses immediately after both pNMES and NMES+. This is similar to recent results derived by Scalia et al. (2023) [41], who investigated the effects of pNMES and NMES+ in older individuals. The authors attributed the lack of modulation of the H-reflex to a different ability of older adults to modulate PSI with respect to young individuals [66,67,68,69,70]. Therefore, the results of our study suggest that in patients with MS, there may also be a change in PSI mechanisms. PSI occurs when an inhibitory neurotransmitter, like gamma-aminobutyric acid (GABA), acts on the GABA receptors of sensory afferent axons, resulting in a reduction in neurotransmitter release. Wang and colleagues (2006a, 2009) [71,72] suggested that changes in GABAergic function may play a key role in producing the motoneuron plasticity, which directly underlies the modulation of the H-reflex after conditioning NMES interventions in healthy individuals. However, it has been demonstrated that the synthesis, release, and reuptake of GABA are altered in MS patients, with GABAergic synapses being more vulnerable to phagocytosis [73,74]. Several findings from transcriptomic, proteomic, neurophysiological, and histological studies indicate that MS is characterized by a pathological alteration of the synaptic structure and function, also known as synaptopathy [74]. Particularly, it has been shown that neuroinflammation perturbates both inhibitory (mediated by GABA) and excitatory (mediated by glutamate) neurotransmission, which are significantly involved in the correct functioning of the CNS. The release of proinflammatory cytokines during acute MS attack increases glutamate-mediated synaptic transmission and reduces γ-aminobutyric acid-mediated synaptic signaling, altering the balance between the GABAergic and glutamatergic systems in the brain and spinal cord. When the alteration of synaptic homeostasis is maintained over time, it can become detrimental, leading to neurodegeneration of the CNS. These observations may be highly relevant in explaining our results, as inhibitory synapses are crucial for healthy neurotransmission [75,76]. However, further investigation is required to elucidate whether and how NMESs alter synaptic neurotransmission in pwMS.

Another factor that may affect the lack of H-reflex response to NMES in MS patients is represented by spinal cord abnormalities. Several studies reported extensive demyelination and neuronal loss of both white and grey matter in the spinal cord [77,78,79,80,81,82]. MS patients show a reduction in synaptic spine density in both myelinated and demyelinated neurons, which leads to irreversible spinal symptoms, including alterations in spinal reflexes [79]. Interestingly, the results of our study showed no differences in the H_max_/M_max_ ratio between MS patients and healthy individuals at baseline. Therefore, the different H-reflex responses between MS and CG could be exclusively attributed to the direct effects of passive and superimposed NMES on inhibitory and regulatory mechanisms that are involved in the synaptic neurotransmission at the spinal cord level. Likely, the MS-induced neuronal damage was not severe enough to alter the H-reflex at rest, as we recruited MS patients with a low level of disability (EDSS ≤ 5). However, when the spinal reflex pathway was overloaded with external stimuli, such as NMES, the damage to the CNS could have impaired the inhibitory/excitatory mechanisms involved in the modulation of the H-reflex, potentially explaining the unaltered H-reflex responses that were found in MS patients after the NMES interventions. However, there is a gap in the literature related to the comparison of the H_max_/M_max_ ratio between MS patients and healthy individuals, as the H-reflex has only been investigated as a measure of spasticity [60,83,84,85,86]. Only one study by Cantrell et al. (2022) [52] investigated the H-reflex in pwMS without spasticity, with the aim of comparing its amplitude between limbs and correlate reflex asymmetry with postural control. This highlights the importance of future studies focused on understanding the mechanisms underlying H-reflex modulation in MS patients who do not present spasticity symptoms. In addition, a better stratification of MS patients according to motor versus sensory impairments as well as the presence or absence of spinal cord abnormalities, and their specific location/site, could help enhance our understanding of spinal mechanisms involved in the modulation of spinal excitability after NMES.

Lastly, according to our hypothesis, no significant differences were found in H-reflex response after ISO in both healthy individuals and MS patients. This result is consistent with previous studies [39,41,42], showing unaltered H-reflexes after a protocol of isometric voluntary contractions of the ankle plantar flexor muscles. The lack of modulation in spinal excitability could be attributed to the short duration of our 3 min exercise protocol, as previously discussed [39,41,42].

There are some limitations in our study. First, PSI was not measured, despite its main role in determining spinal excitability changes [42,49,56]. Thus, further studies should be carried out to evaluate the possible implications of PSI for the different reflex responses between healthy individuals and MS patients following NMES exercise. Second, implementing a longer training protocol could be necessary to observe changes in H-reflex amplitude in pwMS after passive or superimposed NMES, as MS alters some CNS structures and mechanisms that are mainly involved in spinal reflex modulation. In the present study, each experimental condition lasted approximately 3 min. Therefore, our protocol may have been too short to induce acute changes in the spinal circuitry function in MS patients compared to healthy individuals. Nevertheless, with long-term intervention protocols, the effect of muscle fatigue on H-reflex should be taken into account, as muscle fatigue reduces Ia-afferent excitation or increases nerve fiber excitability thresholds [87]. In this regard, we can state that muscle fatigue did not occur as MVIC values did not change between baseline levels and those measured at the end of the entire protocol in both MS and CG [39,49,56]. Finally, another limitation of our study was that our protocol did not allow us to explore the long-term effects of NMES on H-reflex responses. Further longitudinal studies should be designed in a larger cohort of participants with the aim of investigating the chronic effect of NMES interventions on spinal excitability in both healthy individuals and MS patients. These future findings could be of primary importance for elucidating the neurophysiological factors underlying different H-reflex responses in healthy individuals and pwMS in response to chronic interventions. Additionally, from a clinical point of view, these findings may provide evidence for targeted rehabilitation interventions in MS patients.

## 5. Conclusions

The present study demonstrated that MS patients and healthy individuals showed different acute modulations of soleus H-reflex responses after passive and superimposed NMES. While healthy participants showed an acute potentiation of the H-reflex after NMES+ and an attenuation after pNMES, MS patients did not show any changes in the H-reflex amplitudes after both NMES+ and pNMES. This result could be explained by an alteration in the control of some presynaptic mechanisms, involving PSI and GABA release, which are compromised in MS as well as by spinal cord abnormalities that could alter the modulation of the spinal reflex pathway. Moreover, in both MS patients and healthy individuals, the lack of H-reflex responses after ISO indicates that voluntary isometric contractions do not acutely affect spinal excitability compared to pNMES and NMES+. Of practical importance, in MS patients, this result suggests that NMES acts similarly to voluntary isometric exercise, and does not affect spinal excitability. Future studies are warranted to investigate possible spinal-related adaptations and supraspinal modifications as a result of NMES in MS.

## Figures and Tables

**Table 1:** Patients’ data. EDSS, VAS and MAS scores as well as the presence or absence of spinal cord lesion are reported for each participant.

Participant	EDSS	VAS	MAS	Spinal Lesion
1	3	0	0	no
2	1.5	0	0	yes
3	1.5	0	0	no
4	1	0	0	no
5	1	0	0	no
6	1	1	0	yes
7	1	0	0	yes
8	1	0	0	yes
9	1	0	0	yes
10	1.5	0	0	no
11	2	1	0	yes
12	3	0	0	no
13	3	0	0	yes
14	1	0	0	yes
15	1.5	1	0	no
16	2	1	0	yes
17	1	0	0	yes
18	1.5	0	0	no
19	2	1	0	no
20	2.5	0	0	yes

**Table 2:** PRE and POST H-reflex values. The amplitudes of the H-reflex before (PRE) and after (POST) all three experimental conditions (ISO, pNMES, NMES+) are reported as a mean ± standard deviation. The corresponding *p*-values of each condition are illustrated in the table. * Significantly different from PRE.

		PRE	POST	*p*-Value
ISO	MS	0.39 ± 0.29	0.38 ± 0.28	0.506
	CG	0.50 ± 0.24	0.50 ± 0.27	0.829
pNMES	MS	0.42 ± 0.34	0.39 ± 0.32	0.068
	CG	0.49 ± 0.22	0.40 ± 0.20 *	0.000
NMES+	MS	0.40 ± 0.34	0.39 ± 0.31	0.126
	CG	0.48 ± 0.22	0.54 ± 0.25 *	0.010

**Table 3:** Maximal voluntary isometric contraction (MVIC) (Nm). MVIC of ankle plantar flexor muscles before (Pre-test) and after (Post-test) the entire experimental protocol in MS and CG. Data are presented as group means ± standard deviations.

	Pre-Test	Post-Test
MS	43.12 ± 15.1	40.86 ± 15.48
CG	53.43 ± 27.29	54.2 ± 26.45

## Data Availability

The raw data supporting the conclusion of this article will be made available by the authors, without undue reservation.
